# Deaths among Wild Birds during Highly Pathogenic Avian Influenza A(H5N8) Virus Outbreak, the Netherlands

**DOI:** 10.3201/eid2312.171086

**Published:** 2017-12

**Authors:** Erik Kleyheeg, Roy Slaterus, Rogier Bodewes, Jolianne M. Rijks, Marcel A.H. Spierenburg, Nancy Beerens, Leon Kelder, Marjolein J. Poen, Jan A. Stegeman, Ron A.M. Fouchier, Thijs Kuiken, Henk P. van der Jeugd

**Affiliations:** Dutch Center for Avian Migration and Demography, Wageningen, the Netherlands (E. Kleyheeg, H.P. van der Jeugd);; Sovon, Dutch Center for Field Ornithology, Nijmegen, the Netherlands (R. Slaterus);; Utrecht University, Utrecht, the Netherlands (R. Bodewes, J.A. Stegeman);; Dutch Wildlife Health Center, Utrecht (J.M. Rijks);; Netherlands Food and Consumer Product Safety Authority, Utrecht (M.A.H. Spierenburg);; Wageningen Bioveterinary Research, Lelystad, the Netherlands (N. Beerens);; Staatsbosbeheer, Amersfoort, the Netherlands (L. Kelder);; Erasmus Medical Center, Rotterdam, the Netherlands (M.J. Poen, R.A.M. Fouchier, T. Kuiken)

**Keywords:** birds, viruses, outbreaks, die-off, communicable diseases, emerging, ducks, epidemiology, influenza A virus, influenza in birds, mortality, death, population dynamics, poultry, highly pathogenic avian influenza, HPAI, H5N8, tufted duck, Eurasian wigeon, the Netherlands, Holland, influenza

## Abstract

During autumn–winter 2016–2017, highly pathogenic avian influenza A(H5N8) viruses caused mass die-offs among wild birds in the Netherlands. Among the ≈13,600 birds reported dead, most were tufted ducks (*Aythya fuligula*) and Eurasian wigeons (*Anas penelope*). Recurrence of avian influenza outbreaks might alter wild bird population dynamics.

Since 1996, highly pathogenic avian influenza (HPAI) A viruses of the A/goose/Guangdong/1/96 lineage have caused major losses in the poultry industry worldwide and ≈800 confirmed human cases with a mortality rate of ≈50% (*1*,*2*). Wild waterbirds, the natural reservoir of low pathogenicity avian influenza viruses, are probably involved in long-distance spread of HPAI viruses (*3**,**4*).

In May–June 2016, a novel reassortant of HPAI virus subtype H5N8 clade 2.3.4.4a was detected in diseased waterbirds in China (*5*) and on the border between Russia and Mongolia (*6*). In October 2016, a similar H5N8 strain was found in a dead mute swan (*Cygnus olor*) in Hungary (*7*). H5N8 viruses then spread rapidly across Europe, causing widespread death among wild waterbirds (*8*).

## The Study

Concurrent with first detections in Austria, Germany, and Denmark, an H5N8 outbreak started in the Netherlands in early November 2016 (*7*). Unlike previous H5N8 outbreaks in the Netherlands during 2014–2015 (*9**,**10*), increased deaths among wild birds were observed this time.

To quantify deaths among species groups with known susceptibility (*4**,**11*) or that tested positive for H5N8 during the outbreak, we assembled daily mortality data from organizations gathering death reports or removing carcasses in the Netherlands during November 2016–January 2017 (Technical Appendix 1 Table 1). This collection was facilitated by close cooperation between ornithologists, virologists, animal health organizations, and other organizations involved in managing the H5N8 outbreak. After potential double-counts were excluded as much as possible, ≈13,600 wild birds of 71 species were reported dead (Table); 49% of all carcasses were identified by species, most of which were tufted duck (*Aythya fuligula* [39%]) and Eurasian wigeon (*Anas penelope* [37%]). Unidentified waterbird carcasses probably also mostly represented these species. H5N8 infection was confirmed in 21 species and not detected among the low numbers of sampled birds representing 13 other species (Technical Appendix 1 Table 2).

**Table:** Reported bird species, winter population size estimates, number of carcasses, and rRT-PCR test results per incident during outbreak of HPAI A(H5N8) virus, the Netherlands, November 2016–January 2017*

Avian family and species (common name)	Maximum estimated winter population, ×1,000†	No. carcasses	HPAI incidents tested‡
Anatidae (waterbirds)		7,326	51/134
*Anas penelope *(Eurasian wigeon)	680–920	2,511	18/18
*Aythya fuligula* (tufted duck)	190–230	2,633	8/11
Unidentified waterfowl		1,771	23/95
Podicipedidae (grebes)		31	3/5
Ardeidae (herons)§		165	0/13
Phalacrocoracidae (comorants)		50	1/2
Rallidae (rallids)		279	1/9
Scolopacidae (shorebirds)¶		103	0/2
Laridae (gulls)		698	12/28
*Larus marinus* (great black-backed gull)	7.4–13	78	5/5
Accipitridae (hawks)		119	4/17
Falconidae (falcons)		23	3/4
*Falco peregrinus* (peregrine falcon)	0.36–0.52	16	3/4
Corvidae (corvids)		88	3/10
Aves indet. (unidentified)		4,708	4/28
Total		13,590	84/255

*Incidents are defined as death reports of a species per site per day. HPAI, highly pathogenic avian influenza; rRT-PCR, real-time reverse transcription PCR. †Population estimates represent the lowest and highest yearly maxima for the Netherlands during 2009–2014. Data from Sovon (Dutch Center for Field Ornithology, Nijmegen, the Netherlands). ‡Number of positive versus all tested incidents are presented (number positive/number tested), based on infection data from the Netherlands Food and Consumer Product Safety Authority, Dutch Wildlife Health Center, Wageningen Bioveterinary Research, and Erasmus Medical Center.  §H5N8 HPAI virus infection in grey heron (*Ardea cinerea*) was confirmed elsewhere in Europe (*8*).  ¶Eurasian woodcocks (*Scolopax rusticola*) are more prone than other species to window collisions during nocturnal migration; thus, their deaths (54 carcasses reported) might not be related to HPAI.

**Figure 1:**
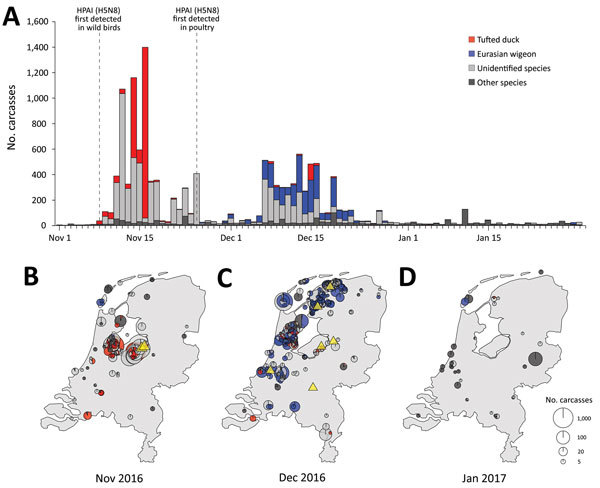
Spatiotemporal pattern of wild bird deaths during an outbreak of HPAI A(H5H8) virus, the Netherlands, November 2016–January 2017. A) Outbreak chronology in tufted duck (red); Eurasian wigeon (blue); unidentified carcasses (light gray), probably also mostly tufted duck and Eurasian wigeon; and all other species combined (dark gray). Dashed vertical lines depict the first detections in wild birds and in poultry in the Netherlands. B–D) Spatial overview of the reported cumulative number of deaths in November 2016 (B), December 2016 (C), and January 2017 (D). Each point on the maps is a pie chart giving the proportions of the respective species (groups) at that location and their size is scaled to the log_10_ of the number of reported carcasses. Yellow triangles mark the locations of outbreaks in commercial poultry holdings. Only locations where >3 dead birds were reported are shown. HPAI, highly pathogenic avian influenza.

After the first H5N8 detection in diseased waterbirds on November 8, hundreds of carcasses were found at Gouwzee (52°27′09′′N, 5°04′07′′E) and Wolderwijd (52°20′51′′N, 5°34′20′′E). Deaths at these locations peaked within 10 days, with ≈5,300 carcasses reported by November 18 (Figure 1). An estimated 85% were tufted ducks. Other species found dead during this period included common pochard (*Aythya ferina* [6%]) and Eurasian coot (*Fulica atra* [4%]), in addition to great crested grebe (*Podiceps cristatus*), mute swan, greater scaup (*Aythya marila*), and several goose and gull species (each <1%).

Beginning in late November, outbreak hotspots moved from open water to water-rich agricultural areas (Figure 1; Video). Deaths predominantly among Eurasian wigeon were reported from the island of Texel (≈883 birds) and the provinces Friesland (≈2,371), Noord-Holland (≈1,375), and Zuid-Holland (≈732). Reports of dead gulls, raptors and corvids, presumably infected after scavenging on carcasses, also increased.

**Video:**
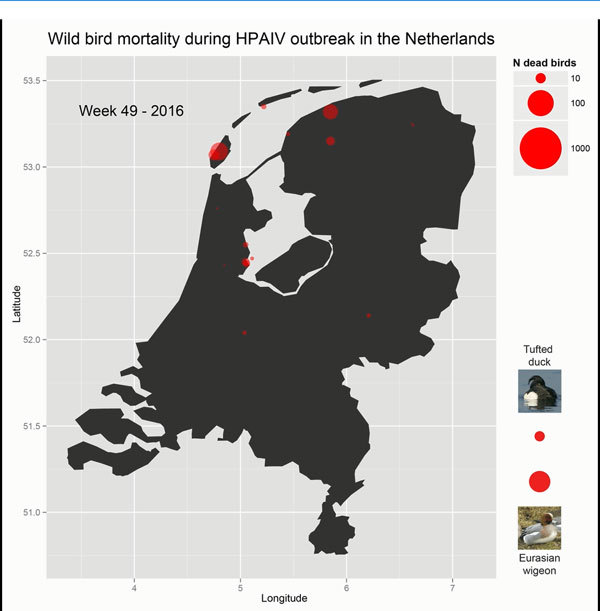
Animated graphic of the weekly progression of an outbreak of highly pathogenic avian influenza A(H5N8) virus, the Netherlands, November 2016–January 2017 (video forthcoming).

Because these data are based on numbers of reported carcasses, they provide an underestimation of actual deaths. Although carcass detection rates during daily searches at Gouwzee and Wolderwijd were estimated to be 90%–95% (C. Oshaar, pers. comm., June 12, 2017), search efficiency was probably much lower at other outbreak hotspots. Collection rates of waterbird carcasses during typical avian botulism outbreaks are 10%–25% (*12*), suggesting that the number of carcasses reported during this H5N8 outbreak represented a limited proportion of total deaths.

We screened a relatively small proportion of carcasses for HPAI virus by real-time reverse transcription on tracheal and cloacal swab samples. We then determined pathogenicity and N-subtype by sequencing, as previously described (*9**,**13*). Testing confirmed H5N8 infection in a large proportion of sampled tufted ducks, Eurasian wigeons, gulls, raptors, and corvids (Table); another HPAI virus subtype was detected only twice (H5N5 in tufted duck and mute swan). 

**Figure 2:**
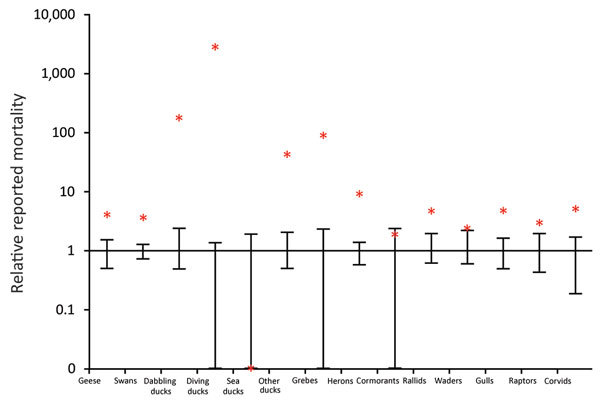
Relative number of deaths among wild birds during an outbreak of highly pathogenic avian influenza A(H5N8) virus, the Netherlands, November 2016–January 2017. Number of reported deaths during November 2016–January 2017 (red asterisks) is shown relative to the normalized number of deaths reported over the same timeframe in the previous 5 years (average is 1, error bars indicate maximum and minimum from 2011–2012 to 2015–2016). The y-axis is on a log-scale (e.g., reported deaths among diving ducks during 2016–2017 was >2,000 times greater than the average reported in the previous 5 years). Within species groups, numbers of deaths are averaged over species. Data from Sovon (Dutch Center for Field Ornithology, Nijmegen, the Netherlands). A graph of the same analysis based on data from the Nature Information Foundation (http://www.waarneming.nl) was also plotted (online Technical Appendix 2, https://wwwnc.cdc.gov/EID/article/23/12/17-1086-Techapp2.pdf).

We used the public science database of Sovon (Dutch Center for Field Ornithology, Nijmegen, the Netherlands) to compare the number of deaths per species group during November 2016–January 2017 with those occurring in the same timeframe from 2010–2011 to 2015–2016 (Figure 2). Death counts among diving ducks (including tufted ducks) were >2,000 times higher than average during November 2016–January 2017, and the relative prevalence of deaths substantially increased (4–177 times) for dabbling ducks, herons, geese, swans, and corvids. The same analysis based on another database (http://www.waarneming.nl) yielded similar results (Technical Appendix 2 Figure).

The elevated number of deaths among wild birds raises concern about potential population effects. After accounting for detection probability (*12*), we found that up to 5% of the wintering populations of tufted ducks and Eurasian wigeons in the Netherlands might have died. In addition, 2%–10% of the wintering population of great black-backed gulls (*Larus marinus*) and 11%–39% of the wintering population of peregrine falcons (*Falco peregrinus*) were similarly affected. Stronger effects were observed locally. At Gouwzee, ≈6,000 tufted ducks were counted in December after ≈2,000 of them had died in November. Assuming that no migration occurred, we estimate that up to 25% of the local population of tufted ducks might have died, which might affect population dynamics substantially. Additional studies are needed to evaluate long-term impacts on these populations and to elucidate why high numbers of birds survived or escaped infection.

The first H5N8 outbreak among poultry in the Netherlands occurred on November 25, two weeks after the first detection in wild waterbirds and coinciding with increasing death reports in Eurasian wigeons. This time lag might be related to the limited mobility of wintering *Aythya* ducks, which, in contrast to Eurasian wigeons, rarely fly over land between foraging and roosting sites. Wild bird ecology might thus affect infection risk among poultry, which was further explored by researchers using network analyses of virus sequences obtained from wild birds and poultry during the same outbreak (*14*).

The quality of reporting of wild bird deaths during this H5N8 outbreak was vastly improved compared with earlier outbreaks, when species names, death rates, and spatiotemporal patterns of deaths were rarely recorded. However, documentation and management of future outbreaks in wild birds can be further improved. To contain outbreaks and minimize losses in the poultry sector, early HPAI virus detection in wild birds is crucial. Monitoring of wild bird deaths can be optimized (e.g., by timely investigation at sites where migratory birds first arrive, especially when surrounding countries report outbreaks). Awareness of clinical signs in wild birds (Technical Appendix 2) might facilitate this effort. Detailed, real-time, active and passive surveillance during outbreaks might help assess acute risk for infection in poultry. Such surveillance would require central coordination of information exchange during outbreaks, which would also facilitate evaluation afterward.

Readily available specific guidelines would help management of HPAI virus outbreaks in wild birds. National HPAI preparedness plans should include specific protocols about how to handle carcasses (e.g., biosafety and disposal instructions) and what to report (e.g., species, number of birds, demographic parameters, and presence of leg bands). Moreover, sufficient resources should be available for adequate sampling and testing of specimens to rule out other diseases and to track virus dynamics during an outbreak.

## Conclusions

Our findings indicate that the 2016–2017 H5N8 outbreaks in the Netherlands were associated with unprecedented high HPAI-related mortality rates in a wide range of wild bird species. These latest H5N8 outbreaks have shifted the paradigm of wild birds as unaffected agents of HPAI viruses, with increasing concerns about potential effects on their populations. The Netherlands and other important staging areas for migratory waterbirds across Eurasia that have been affected by the 2016–2017 H5N8 outbreaks (*3**,**15*) are at risk for substantial numbers of bird deaths during future HPAI outbreaks. International responsibilities regarding migratory bird populations should stimulate national authorities to avert HPAI outbreaks not only in poultry and humans but also in wild birds.

Technical Appendix 1 Overview of organizations and authorities that provided data for analysis of wild bird deaths and a full species account of reported cases of avian deaths during an outbreak of highly pathogenic avian influenza A virus subtype H5N8, the Netherlands, November 2016–January 2017.

Technical Appendix 2 Relative number of deaths among wild birds, based on data from the Nature Information Foundation and an overview of clinical signs in wild birds reported during an outbreak of highly pathogenic avian influenza A virus subtype H5N8, the Netherlands, November 2016–January 2017.

## References

[1] Lai S, Qin Y, Cowling BJ, Ren X, Wardrop NA, Gilbert M, et al. Global epidemiology of avian influenza A H5N1 virus infection in humans, 1997-2015: a systematic review of individual case data. Lancet Infect Dis. 2016;16:e108–18. 10.1016/S1473-3099(16)00153-5 27211899 PMC4933299

[2] World Health Organization. Cumulative number of confirmed human cases of avian influenza A(H5N1) reported to WHO [cited 2017 June 12]. http://www.who.int/influenza/human_animal_interface/H5N1_cumulative_table_archives

[3] Keawcharoen J, van Riel D, van Amerongen G, Bestebroer T, Beyer WE, van Lavieren R, et al. Wild ducks as long-distance vectors of highly pathogenic avian influenza virus (H5N1). Emerg Infect Dis. 2008;14:600–7. 10.3201/eid1404.071016 18394278 PMC2570914

[4] Lycett SJ, Bodewes R, Pohlmann A, Banks J, Bányai K, Boni MF, et al.; Global Consortium for H5N8 and Related Influenza Viruses. Role for migratory wild birds in the global spread of avian influenza H5N8. Science. 2016;354:213–7. 10.1126/science.aaf8852 27738169 PMC5972003

[5] Li M, Liu H, Bi Y, Sun J, Wong G, Liu D, et al. Highly pathogenic avian influenza A(H5N8) virus in wild migratory birds, Qinghai Lake, China. Emerg Infect Dis. 2017;23:637–41. 10.3201/eid2304.161866 28169827 PMC5367427

[6] Lee DH, Sharshov K, Swayne DE, Kurskaya O, Sobolev I, Kabilov M, et al. Novel Reassortant clade 2.3.4.4 avian influenza A(H5N8) virus in wild aquatic birds, Russia, 2016. Emerg Infect Dis. 2017;23:359–60. 10.3201/eid2302.161252 27875109 PMC5324796

[7] World Organisation for Animal Health (OIE). Avian influenza portal [cited 2017 May 29]. http://www.oie.int/animal-health-in-the-world/update-on-avian-influenza

[8] More S, Bicout D, Botner A, Butterworth A, Calistri A, et al. Urgent request on avian influenza. EFSA J. 2017;15:4687. 10.2903/j.efsa.2016.4687 PMC7009852 32625275

[9] Verhagen JH, van der Jeugd HP, Nolet BA, Slaterus R, Kharitonov SP, de Vries PP, et al. Wild bird surveillance around outbreaks of highly pathogenic avian influenza A(H5N8) virus in the Netherlands, 2014, within the context of global flyways. Euro Surveill. 2015;20:21069. 10.2807/1560-7917.ES2015.20.12.21069 25846491

[10] Poen MJ, Verhagen JH, Manvell RJ, Brown I, Bestebroer TM, van der Vliet S, et al. Lack of virological and serological evidence for continued circulation of highly pathogenic avian influenza H5N8 virus in wild birds in the Netherlands, 14 November 2014 to 31 January 2016. Euro Surveill. 2016;21:30349. 10.2807/1560-7917.ES.2016.21.38.30349 27684783 PMC5073202

[11] Breed AC, Harris K, Hesterberg U, Gould G, Londt BZ, Brown IH, et al. Surveillance for avian influenza in wild birds in the European Union in 2007. Avian Dis. 2010;54(Suppl):399–404. 10.1637/8950-053109-Reg.1 20521669

[12] Bollinger TK, Evelsizer DD, Dufour KW, Soos C, Clark RG, Wobeser G, et al. Ecology and management of avian botulism on the Canadian prairies [cited 2017 Jun 20]. http://www.phjv.ca/pdf/BotulismReport_FINAL_FullReport_Aug2011.pdf

[13] Bouwstra RJ, Koch G, Heutink R, Harders F, van der Spek A, Elbers ARW, et al. Phylogenetic analysis of highly pathogenic avian influenza A(H5N8) virus outbreak strains provides evidence for four separate introductions and one between-poultry farm transmission in the Netherlands, November 2014. Euro Surveill. 2015;20:21174. 10.2807/1560-7917.ES2015.20.26.21174 26159311

[14] Beerens N, Heutink R, Bergervoet SA, Harders F, Bossers A, Koch G. Multiple reassorted viruses as cause of highly pathogenic avian influenza virus A(H5N8) epidemic, the Netherlands, 2016. Emerg Infect Dis. 2017;23:1974–81. 29148396 10.3201/eid2312.171062 PMC5708218

[15] Pohlmann A, Starick E, Harder T, Grund C, Höper D, Globig A, et al. Outbreaks among wild birds and domestic poultry caused by reassorted influenza A (H5N8) clade 2.3. 4.4 viruses, Germany, 2016. Emerg Infect Dis. 2017;23:633–6. 10.3201/eid2304.161949 28055819 PMC5367393

