# Supplementary material for: Deaths among Wild Birds during Highly Pathogenic Avian Influenza A(H5N8) Virus Outbreak, the Netherlands
Source: Emerg Infect Dis. 2017 Dec;23(12):2050–4. doi: 10.3201/eid2312.171086 (PMC5708256; doi:10.3201/eid2312.171086)
Supplement: Technical Appendix 2 — Relative number of deaths among wild birds, based on data from the Nature Information Foundation and an overview of clinical signs in wild birds reported during an outbreak of highly pathogenic avian influenza A virus subtype H5N8, the Netherlands, November 2016–January 2017. [file 17-1086-Techapp-s2.pdf]

Article DOI: https://doi.org/10.3201/eid2312.171086

Deaths among Wild Birds during Highly Pathogenic Avian Influenza A(H5N8) Virus Outbreak, the Netherlands

Technical Appendix 2

Overview of Clinical Signs Reported in Wild Birds in Association with Mortality during the H5N8 Outbreak in the Netherlands, November 2016–January 2017

Throughout the H5N8 outbreak period, fatal cases were regularly reported along with a description of pre-mortal disease signs that were readily identified by the (usually lay) observer. Waterbirds generally showed either one of two types of signs. Eurasian teal *Anas crecca* (1 out of 1), Eurasian wigeon *Anas penelope* (2/4), mallard *Anas platyrhynchos* (1/1), tufted duck *Aythya fuligula* (6/6), greylag goose *Anser anser* (1/1), feral goose *Anser anser* forma *domesticus* (1/1) and mute swan *Cygnus olor* (1/2) made a severely weakened impression, sometimes accompanied with signs of paralysis, apathy and unresponsiveness to threats like human approach or dog attack. Tufted ducks retreated into shoreline vegetation. A mute swan had respiratory problems. Other waterfowl (2/4 Eurasian wigeons, 4/4 unidentified ducks, probably also Eurasian wigeons, and 1/2 mute swan) showed clinical signs consistend with neurologic disease: writhing, rocking their body or swimming and walking in circles, consistent with neurologic disease. A common coot *Fulica atra* appeared to die without showing prior signs of illness or distress, while another common coot, a great cormorant *Phalacrocorax carbo* (1/1) and a great egret *Ardea alba* (1/1) appeared severely weakened before death. One unidentified gull was seen falling from the sky and dying within several seconds, which is consistent with multiple reports of dead unidentified gulls that appeared to have dropped out of the sky based on their unusual finding locations. Raptors were all weakened and approachable (9/9 common buzzards *Buteo buteo* and 3/3 peregrine falcons *Falco peregrinus*). Five common buzzards and one peregrine falcon appeared to have lost their balance, twisting their head or walking in circles. In one common buzzard and two peregrine falcons blood was coming out of the bill. Corvids

showed a variety of signs, from only apathetic (1/1 Western jackdaw *Corvus monedula*), to moaning (1/2 Eurasian magpies *Pica pica*), mucus (1/2 Eurasian magpies), imbalance and twisting the head (1/2 unidentified corvids) and respiratory distress and diarrhea (1/2 unidentified corvids). In most cases, death followed within an hour after the bird was first observed, but it is unclear whether signs were present earlier. It should also be noted that, although all above cases occurred during the H5N8 outbreak period, infection with H5N8 was generally not tested for these specific individuals.

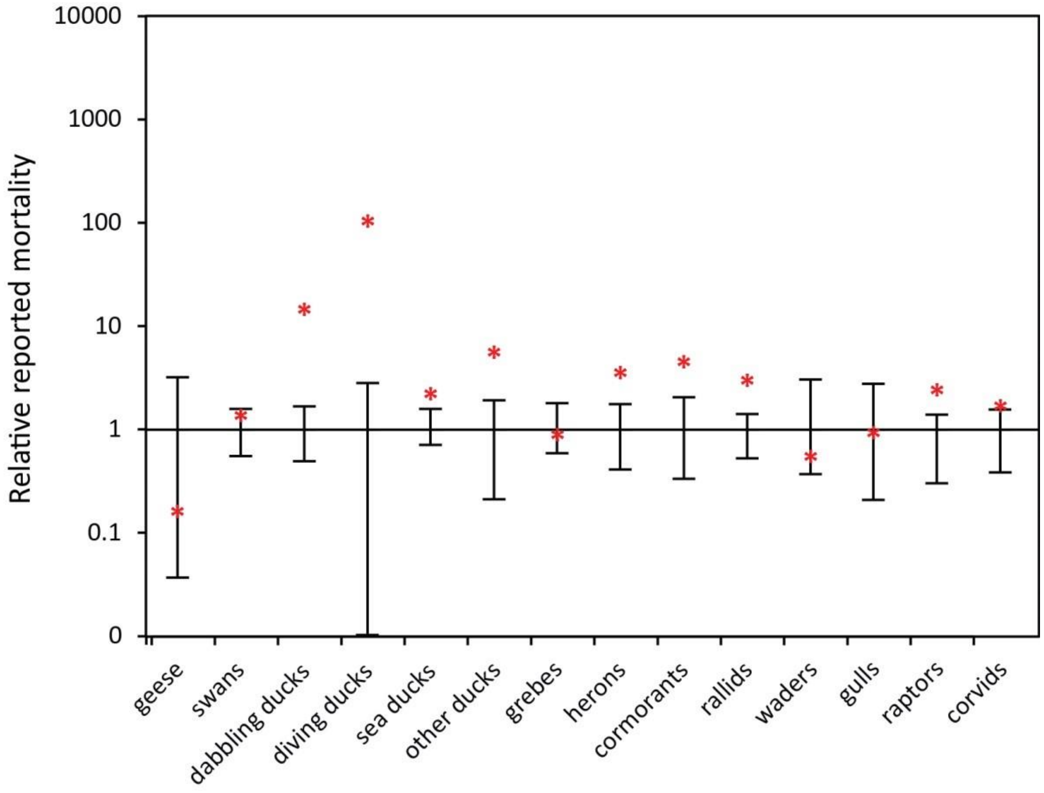

Technical Appendix 2 Figure. Relative number of deaths among wild birds during an outbreak of highly pathogenic avian influenza A virus subtype H5N8, the Netherlands, November 2016–January 2017. Number of reported deaths in November 2016–January 2017 (red asterisks) relative to the normalized number of deaths reported over the same time frame in the previous 5 years (average is 1, error bars indicate maximum and minimum from 2011/2012 to 2015/2016). Within species groups, numbers of deaths are averaged over species. Note that the y-axis is on a log-scale. Data from the citizen science database the Nature Information Foundation (www.waarneming.nl). A graph of the same analysis based on data from Sovon (Dutch Center for Field Ornithology), is presented in the main article.
